# Soil Fertilization Leads to a Decline in Between-Samples Variability of Microbial Community δ^13^C Profiles in a Grassland Fertilization Experiment

**DOI:** 10.1371/journal.pone.0044203

**Published:** 2012-09-04

**Authors:** Stavros D. Veresoglou, Barry Thornton, George Menexes, Andreas P. Mamolos, Demetrios S. Veresoglou

**Affiliations:** 1 Laboratory of Ecology and Environmental Protection, Faculty of Agriculture, Aristotle University of Thessaloniki, Thessaloniki, Greece; 2 Environmental and Biochemical Sciences Group, The James Hutton Institute, Craigiebuckler, Aberdeen, United Kingdom; 3 Laboratory of Agronomy, School of Agriculture, Aristotle University of Thessaloniki, Thessaloniki, Greece; 4 Plant Ecology, Freie Universität Berlin, Berlin, Germany; U. S. Salinity Lab, United States of America

## Abstract

Gas chromatography combustion isotope ratio mass spectrometry (GC-C-IRMS) was used to measure the ^13^C/^12^C ratios of PLFAs at natural abundance levels from a temperate grassland nitrogen (N) and phosphorus (P) factorial fertilization experiment in northern Greece. In each plot two rhizosphere samples were derived centred around individual *Agrostis capillaris* and *Prunella vulgaris* plants. It was hypothesized that the isotopic signal of microbes that preferentially feed on recalcitrant litter such as fungi would be modified by fertilization more strongly than that of opportunistic microbes using labile C. Microbial community δ^13^C was affected by both P and N fertilization regime and plant species identity. However, we have been unable to detect significant nutrient effects on individual groups of microbes when analyzed separately in contrast to our original hypothesis. Intra-treatment variability, as evaluated from Hartley’s *F*_max_ tests in the five first PCA components axes as well as the size of the convex hulls in PCA scoreplots and Mahalanobis distances, was considerably higher in the non-fertilized controls. Moreover, a significant relationship was established between the change in PLFA abundances and their respective changes in δ^13^C for the aggregate of samples and those simultaneously fertilized with N and P. We conclude that use of compound specific isotope analysis in the absence of labelling represents a valuable and overlooked tool in obtaining an insight of microbial community functioning.

## Introduction

Carbon (C) isotopes represent a diagnostic tool for soil ecosystem functioning [1]. Carbon occurs in nature in the form of three isotopes, ^12^C, ^13^C, ^14^C at abundances 98.89%, 1.1% and 0.01% respectively [2]. In isotope studies at natural abundance levels the ^13^C content of a sample is usually measured through the δ^13^C value defined as: δ^13^C (‰) = 1000×(R_sample_ – R_standard_)/R_standard_‰, where R_sample_ and R_standard_ are the ^13^C/^12^C abundance ratios of the sample and a reference standard (Vienna-Pee Dee Belemnite) [3]. Compound specific, including phospholipid fatty acids (PLFA), isotope analysis through gas chromatography combustion isotope ratio mass spectrometry (GC-C-IRMS) can provide information of carbon pathways at the molecular scale [4].

The vast majority of soil microbes are heterotrophes and rely on C assimilation to meet their energy needs. Carbon limitation of microbial growth, that is commonly reported in the soil environment [5], results in most microbes aggregating in carbon “hot-spots” such as the rhizosphere [6]. However, assimilation speed of carbon by the microbial community may vary considerably for different carbon compounds [7]. Moreover, for some carbon compounds the range of potential degraders appears to be narrower than others. For example lignin is mainly degraded by white rot fungi; whilst glycine may be assimilated by a narrower range of microbial groups than other exudates [8], [9].

In the short term organic residues in soil exhibit a δ^13^C fractioning as more recalcitrant litter compounds such as waxes and lignin are known to be more depleted (possess more negative δ^13^C) in ^13^C [10], [11], [12]. Wedin et al. [13] were able to demonstrate, in an incubation study, that the proportion of lignin fraction of grass litter in organic matter increased with time and that it was consistently more depleted in ^13^C than the “bulk” of organic matter. However, in contrast to their original hypothesis, the overall isotopic signature of organic matter, δ^13^C, did not change towards that of lignin. This is in agreement with an increasing amount of literature that has shown an increase in ^13^C content (more positive δ^13^C signal) of soil organic matter when organic matter ages [11], [14]. It is now believed that in the longer term, decomposability of soil compounds is determined by accessibility of the substrate to the microbial community and the rate of decomposition is determined by abiotic factors [15], [16]. Selective use of C compounds from microbial groups, along with kinetic fractionation, the isotopic discrimination for carbon during microbial consumption [17], are perceived to be the two mechanisms that shape the isotopic composition of microbially respired CO_2_ that may differ considerably from that of the organic matter [18]. Thereby, isotopic δ^13^C signal of bulk tissue of microbial groups, that is believed to be depleted at a scale of 0.7–2.8‰ relative to respired CO_2_ [19], may reflect that of the compounds been assimilated. Microbes that feed on older more recalcitrant litter could, consequently, be expected to possess less depleted isotopic δ^13^C bulk tissue. Phospholipids, although further depleted in ^13^C relative to bulk tissue at a scale of 6–8‰ [20], because of the rapid post-death degradation in the soil environment [21] may represent an ideal target to study microbial group δ^13^C signals.

GC-C-IRMS analysis of soil samples has, mainly, been applied following ^13^C feeding of the microbial community [22], ^13^CO_2_ labelling of plants [23] or a shift from C_4_ to C_3_ vegetation [24]. While the specific approach has the potential to provide invaluable information on the microbial groups that utilize the labelled compounds, it may be of more limited applicability in long term studies because spiked δ^13^C signal fade with time and high noise can result from natural variability in δ^13^C of microbial cell membranes. An alternative way to conduct analysis of long-term dynamics of litter could be through GC-C-IRMS analysis of PLFAs at natural abundance levels. A challenge in this case remains the interpretation of natural isotopic signals of individual signatures. To date, natural abundance GC-C-IRMS analysis of PLFAs in soil ecology has been very restricted; the few studies which have adopted this specific approach have made limited attempts to draw inferences out of them [25], [26]. By contrast, natural abundance GC-C-IRMS is routinely applied in marine studies [27].

It is well established that fertilization of soil may evoke considerable shifts with regards to terrestrial C cycling. The traditional view that fertilization may increase decomposition rates [28] has been challenged by Berg and Meentemeyer [29] who argued that at higher N levels (low C:N ratio) more litter remains at the stage when decomposition virtually ceases. In agreement with this view, the microbial N mining hypothesis [30] states that following N addition, rates of decomposition decline as microbes use the readily available N that is applied through fertilization instead of decomposing organic compounds, a hypothesis supported by results from Craine et al. [31]. By contrast, Galantini and Rossell [32] were able to demonstrate that N and phosphorus (P) long term fertilization resulted in an increase of the ratio of labile to recalcitrant organic fractions. While a considerable amount of literature has addressed the impact of fertilization practices in the short-term [33], [34], there is increasing awareness that ecosystem feedback to pulsed nutrient addition on the longer term may differ [35], [36], [37]. The effects of fertilization on C-cycling are of high ecological relevance as they could implicate with the C sequestration ability of soils. Post industrial increase in N deposition had been predicted, through increased primary productivity, to result in increases in C sequestration [38] but empirical results often reveal that more complicated mechanisms may operate [39].

We launched a study of exploratory nature on the potential use of natural abundance GC-C-IRMS as a tool to get an insight of microbial community responses to medium-term (*i.e* 3–10 years) fertilization in a C_3_ grassland in northern Greece. Absence of C_4_ plants, which produce plant litter with distinct δ^13^C signal, restricted ^13^C variability in plant litter and exudates and enabled us to attribute δ^13^C signal of individual microbial groups mainly to isotopic discrimination during photosynthesis, litter decomposition, fractionation and discrimination during C assimilation. We anticipated that despite the natural gradient of δ^13^C that occurs amongst C compounds, the δ^13^C isotope signal would reflect the persistence time in the soil and that the recalcitrant fraction of soil organic matter (older in age) would be characterized by less depleted ^13^C. We, thus, expected that microbial group δ^13^C largely reflected the isotopic signal of the compounds consumed. We primarily wanted to assess the extent of differences in the natural abundance δ^13^C signal of individual microbial groups as affected by the fertilization practices. As stated earlier, fertilization results in longer residence time for the recalcitrant fraction of litter [29], [30], [31], a litter fraction with a δ^13^C signal dependent on decomposition history [11], [14]. We hypothesized that the isotopic signal of microbial groups that specialize on the assimilation of recalcitrant litter such as fungi would have been affected more strongly than the respective δ^13^C of more opportunistic microbial groups such as bacteria that use labile C. We further hypothesized that the microbial groups that would have gained access, following fertilization, to less recalcitrant litter would have increased in relative abundance whereas those that utilised less readily available compounds would have declined. This could be reflected in a linear relationship between change in δ^13^C (Δ^13^C - a capital Δ is used for δ^13^C differences as opposed to isotopic signal where the use of a small δ has been adopted) of individual PLFA signatures (since recalcitrant compounds may be expected to be less depleted in δ^13^C) and relative change in PLFA signature abundance. Further to the ecological significance (to the best of our knowledge the specific question has not been addressed in the past) of recovering a negative relationship between the two variables tested this would additionally confirm the sensitivity of the GC-C-IRMS approach adopted in the manuscript in the absence of labelling as a means of studying the physiology of the microbial community in soils.

## Results

**Figure 1:**
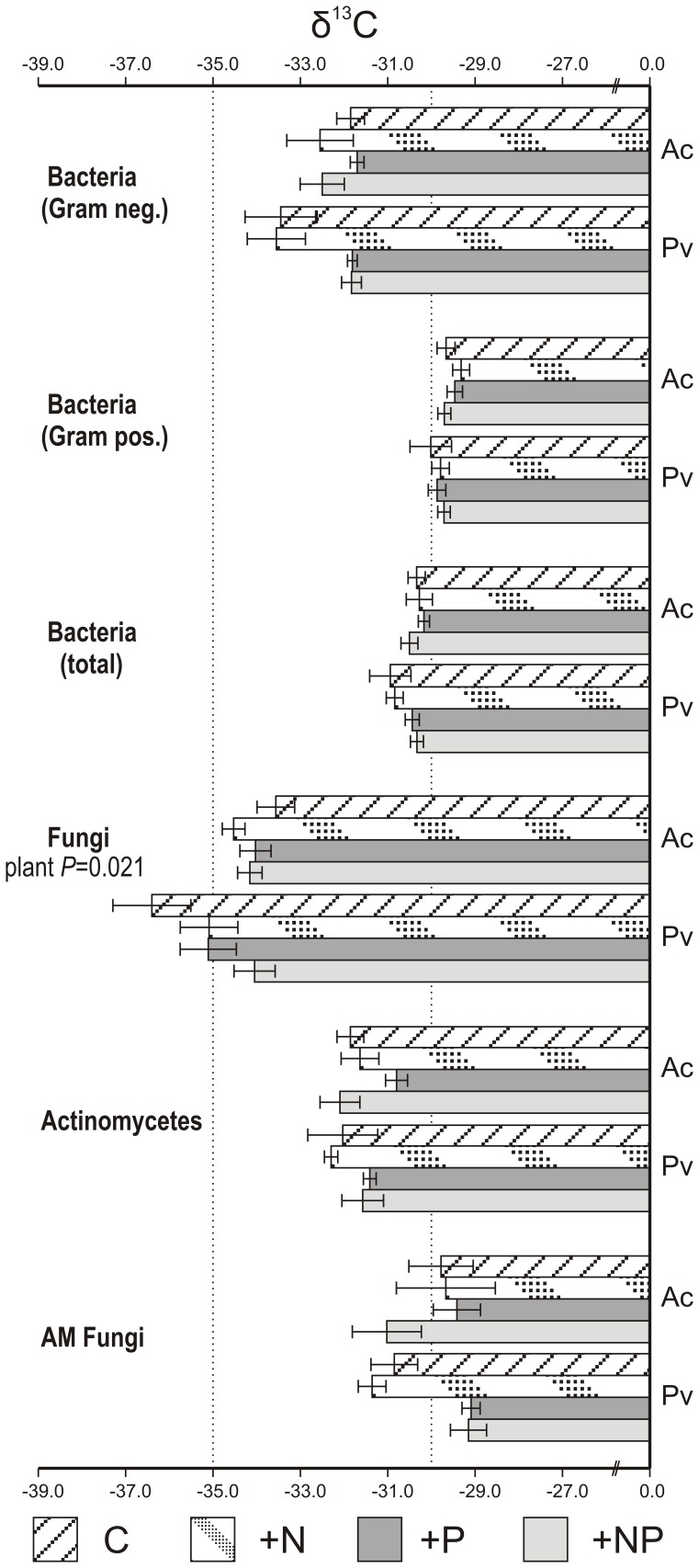
Isotopic signal of key microbial groups. δ^13^C value (±S.E.) of main microbial groups in the fertilization experiment as affected by N and P fertilization practices and plant species identity. The only significant effect was that of plant species on fungi. The two dotted lines have been drawn for reference purposes for δ^13^C signals equal to −30 and −35, respectively. Abbreviations stand for: C-control +N-N fertilized samples +P-P fertilized samples +NP = NP fertilized samples, Ac-*Agrostis capillaris*, Pv-*Prunella vulgaris*.

**Figure 2:**
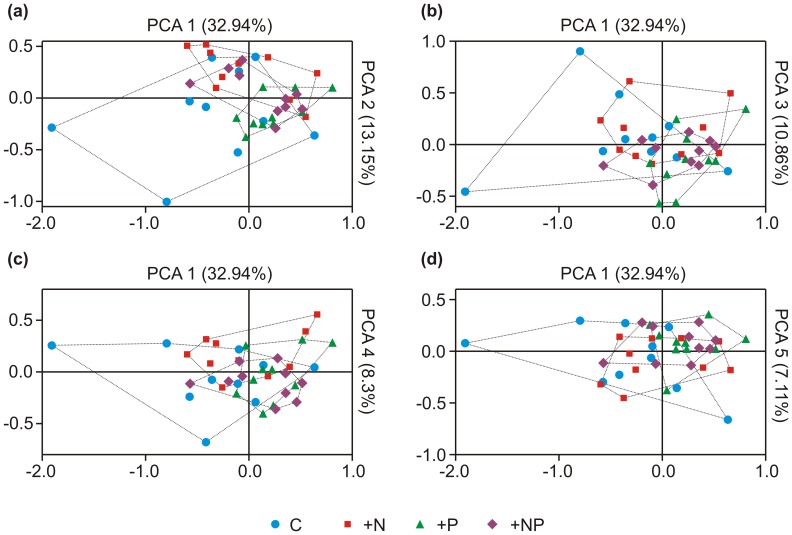
Ordination diagrams of the isotopic signal of PLFA signatures. Principal component analysis distance scoreplots and respective convex hulls of PCA scores grouped according to N and P fertilization regime. In all plots the *x* axis illustrates PCA scores of the first component whereas the *y* axis illustrates PCA scores of the (a) second; (b) third; (c) fourth and (d) fifth component, respectively. The five axes explained a cumulative 72.36% of variance. Abbreviations stand for: C-control +N-N fertilized samples +P-P fertilized samples +NP = NP fertilized samples.

Kolmogorov-Smirnov tests did not detect violations of normality in PLFA δ^13^C signals at a scale that would prohibit PCA. Normality of the microbial group datasets that were used for analysis of variance was also confirmed. The analysis of variance did not reveal significant effects of either the two manipulations (N and P fertilization) or the plant species (rhizosphere) effect in the microbial groups examined with the exception of fungi where a significant plant species effect was detected (*F*_1,28_ = 10.52, *P* = 0.003– after Bonferroni *P* = 0.021) (Fig. 1). However, a trend of P fertilization practice resulting in an increase of δ^13^C value of gram negative bacteria existed (*F*_1,28_ = 5.996, *P* = 0.02– after Bonferroni *P* = 0.14) as well as a trend for an interactive effect of species and P fertilisation on AM fungal δ^13^C value (*F*_1,28_ = 6.532, *P* = 0.016– after Bonferroni *P* = 0.11). The broken stick model revealed significance of the five first PCs that explained a cumulative 72.36% of total variance. PCA distance scoreplots revealed overlap of the imposed treatments (Fig. 2). According to MANOVA analysis, however, the effects of P (*P* = 0.001– significant for the 1^st^, 3^rd^ and 5^th^ principal components) N (*P* = 0.011– significant for the 2^nd^ component) and plant (*P* = 0.001– significant for the 5^th^ principal component) manipulations on δ^13^C values were rendered significant as well as the interactive effect of plant and P (*P* = 0.04– the effect was not significant for any individual component considered) (Table 1).

**Table 1:** Pillai’s Trace statistics following a MANOVA analysis of microbial community δ^13^C PCA scores.

Effect	Pillai’s Trace	*F*	*P*	PCs
Block	0.906	1.580	0.071	
Plant	0.538	5.585	**0.001**	5
N	0.444	3.840	**0.011**	2
P	0.564	6.214	**0.001**	1,3,5
Plant * N	0.293	1.988	0.117	
Plant * P	0.365	2.760	**0.042**	–
N * P	0.317	2.232	0.084	
Plant * N * P	0.199	1.194	0.342	

The broken stick model retrieved five significant PCA components. The column PCs highlights the principal components where significance of the factor has been detected.

We, subsequently, tested violation of the homogeneity of variance criterion in the PCA scores grouped according to N and P manipulations. For all five principal components the *F*_max_ test was significant (*P*<0.05, Table 2). Non-fertilized samples consistently were those where the highest variability with respect to PLFA δ^13^C signal was recorded. Convex hulls that were obtained from combination of the five first principal components verified that non-fertilized samples exhibit the highest variability with respect to δ^13^C signals (Table 3). Finally Mahalanobis distances of individual samples are presented in Table S1. The Kruskal-Wallis test, that explored the impact of the four fertilization treatments on the ranking of the Mahalanobis distances, was significant (*P* = 0.039). We therefore concluded that a significantly higher variance existed in unfertilized plots.

**Table 2:** Variances of scores in the five first PCA component axes of the PCA grouped according to fertilization manipulation and results of the Hartley *F*_max_ test for homogeneity of variances.

	Variance control	Variance N-fertilized	Variance P-fertilized	Variance NP-fertilized	*F* value^a^	*P* a
PCA Comp. 1	0.46	0.20	0.08	0.12	5.93	0.007
PCA Comp. 2	0.18	0.05	0.03	0.04	5.83	0.007
PCA Comp. 3	0.15	0.07	0.07	0.02	6.33	0.006
PCA Comp. 4	0.09	0.04	0.06	0.02	3.56	0.036
PCA Comp. 5	0.10	0.04	0.04	0.03	3.69	0.033

a Values obtained from an *F*_max_ comparison between the non fertilized control and the less variable fertilization treatment.

**Table 3:** Convex hull sizes of samples grouped according to fertilization regime.

PCA Components	Convex hull size control	Convex hull size N-fertilized	Convex hull size P-fertilized	Convex hull size NP-fertilized
1,2	1.95	0.47	0.23	0.35
1,3	1.62	0.76	0.41	0.32
1,4	1.21	0.47	0.38	0.32
1,5	1.06	0.51	0.37	0.33
2,3	0.80	0.34	0.21	0.18
2,4	0.72	0.30	0.27	0.19
2,5	0.69	0.26	0.19	0.22
3,4	0.70	0.31	0.40	0.09
3,5	0.71	0.26	0.34	0.14
4,5	0.54	0.27	0.28	0.14

**Figure 3:**
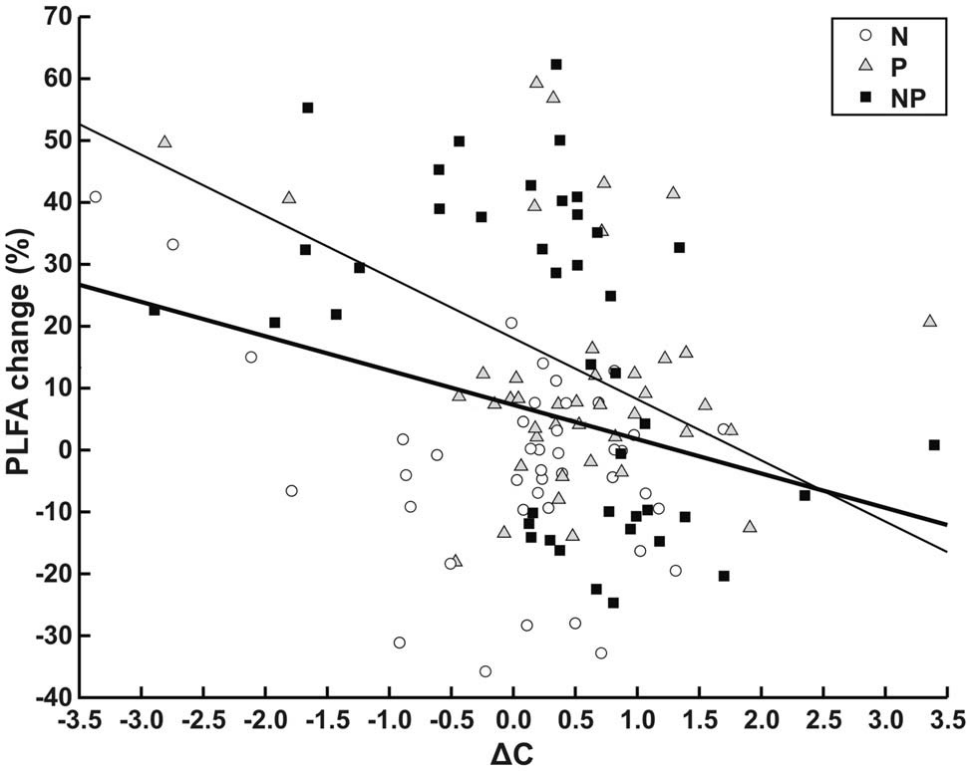
Modification of PLFA abundance vs isotopic signal diagram. Scatter plot of the relationship between percent change of individual PLFA concentrations and Δ^13^C in the fertilized treatments compared to the non-fertilized controls. Open circles: N fertilized samples, triangles: P fertilized and squares: NP fertilized samples. The thin continuous line is the best fit least-squares line for the NP fertilized samples. The thick continuous line is the least-squares trendline for all three fertilization treatments when compared to the control. Note, non-significant trendlines for N and P fertilized samples were not drawn.

Correlation of the weighted difference in concentration with the respective non-fertilized control and ΔC signalling was significant for the NP treatment *(n* = 42, Spearman *ρ* = −0.477, *P*<0.01) as well as for the entire dataset (*n* = 126, Spearman *ρ* = −0.177, *P*<0.05) (Fig. 3).

## Discussion

In C_3_ grasslands a major source of fractionation of the plant community in δ^13^C is the water use efficiency. Isotopic discrimination of ^13^C greater than 22‰ may result during photosynthesis in C_3_ plants [40], [41]. Whilst the majority of this is resultant from metabolic discrimination, it is also in part due to stomatal aperture changes. However, in the long term plants show an intermediate ^13^C fractioning response towards the typical moisture regime of their environment. The upland grassland where the experiment was located was typical Mediterranean semi-arid grassland where thaw of ice in April and drought during summer months result in a short plant growing season. Preliminary experimentation had revealed heterogeneity in water availability between different blocks (Fig. S1) and this could have accounted for the absence of detectable Euclidean space differences in δ^13^C amongst fertilization treatments. On the other hand, persistence of this short growth season signified that temporal complementarity in plant growth was maintained to a minimum [42] and that this could constrain across-years variability with regards to plant litter δ^13^C signal. Ehleringer et al. [11] and Bowling et al. [14] were able to detect a difference in δ^13^C between litter and bulk organic matter deep in soil of approximately 2‰. Fertilization effects may have consequently fallen short of the natural soil C recalcitrance variation on δ^13^C.

MANOVA analysis revealed that P and N fertilization regimes and plant species identity had a significant effect on microbial δ^13^C values (Fig. 2). N fertilization, through altering the water use efficiency (either positively or negatively) of plant species, has been repetitively demonstrated to be a key regulator of δ^13^C signal of the plant community and, consequently, plant litter [43]. In Hobbie and Colpaert [43] N additions through deteriorating water use efficiency resulted in plants grown in N-fertilized plots possessing 1–1.5‰ less depleted (more positive) δ^13^C. The relatively low significance of N fertilization practice on PLFA δ^13^C was, thus, a surprising outcome of the analysis. However, in the specific sampling area P fertilization had a more pronounced impact than N in shaping microbial community clustering patterns [44].

Because of the exploratory nature of the study a conservative approach in interpreting data was adopted. Hence, the influence of different treatments was evaluated following a classical Bonferroni correction. In an earlier analysis of PLFA concentrations [44] no significant responses of the main microbial groups, as a direct effect of either fertilization or plant species identity, was observed. Therefore differences in δ^13^C could not be attributed to microbial community shifts and comparisons were facilitated. The specific approach revealed that the only microbial group whose δ^13^C was significantly (*P*<0.05) affected by manipulations were fungi (AM fungi were separately analyzed and excluded from the fungal group as they assimilate plant derived labile carbon and, thus, exhibit a distinct δ^13^C signal – no ectomycorrhizal plants were present in the grassland – Fig. 1). In specific, *P. vulgaris* appeared to support a fungal population with more depleted ^13^C phospholipids than *A. capillaris* did. Soil fungi are a heterogeneous group of decomposers some of which (e.g. white rot fungi) exhibit high specificity on the carbon compounds they consume. Therefore, significance of the plant identity effect could reflect qualitative differences in litter quality produced that could have altered the relative composition within the fungal group. Alternatively, fungal PLFA signatures have been demonstrated to more readily respond to ^13^CO_2_ labelling which signifies dependence on newly released photoassimilates [45]. Differences in exudation patterns and/or extent of mycorrhizal colonization between the two plants could justify the recorded differences in fungal phospholipid content in ^13^C.

Lower inter-sample variance in δ^13^C of microbial groups was recorded in the fertilized plots. The authors attribute this to microbes having narrowed the range of carbon compounds they assimilated. The N mining hypothesis predicts a decline in the degrading efficiency of the recalcitrant fraction of plant litter following N fertilization. Similarly, evidence suggests that the increase in microbial activity that is known to follow a fertilization event is transient and in the longer term microbial biomass declines [37]. The decline is more intense for fungi that are responsible for degrading the recalcitrant fraction litter [37], [46]. Apparently, following chronic fertilization, the microbial community targets labile C and this makes microbial nutrition more predictable and constrains inter-sample variation in δ^13^C. With regards to the hypothesis that the microbial groups that increased in abundance following fertilization would have utilised less recalcitrant pools of carbon and would be characterized by a decline in δ^13^C (although in Veresoglou et al. [44] the differences that had been detected were not significant) we were able to notice a linear relationship in agreement to the proposed hypothesis. The PLFA signatures that increased in abundance had actually been able to feed in more depleted in ^13^C litter.

As stated in the introduction the specific study represents one of the very few attempts to utilize GC-C-IRMS in the absence of ^13^C labelling in studies addressing soil microbial communities. The authors were able to use this data to report inferences on the inter-sample variability of δ^13^C microbial profiles and the linear relationship between relative shifts in individual signature concentration and Δ^13^C of signal. The main advantage of the approach has been the fact that it is fast to conduct and does not require the extensive optimization procedure that accompanies a ^13^C labelling experiment. By contrast, the main drawback of the method may be its limited discrimination power as a maximum isotopic difference of 4‰ was recorded between microbial groups in the case between fungi and arbuscular mycorrhizal fungi. Such a small discrimination power may lead to a masking of significant effects because of “noise” generated by environmental and analytical variability and further complicate interpretation of data. Additionally, we had to confront the lack of background literature to compare our results to but the specific issue could be resolved following an increase in the number of studies in the near future that utilize this specific approach.

In conclusion, GC-C-IRMS in the absence of ^13^C labelling may represent an interesting tool in evaluating responses of microbial groups to environmental manipulations. The technique suffers from low discrimination power due to low natural fractioning of C compounds but may provide a vehicle to address issues such as natural variability in isotopic composition of microbes between samples and overall patterns of PLFA concentration and Δ^13^C responses to environmental factors.

## Materials and Methods

### Field Site and Experimental Design

The experimental area was located at a high elevation site 160 km west of Thessaloniki in Northern Greece (40o48′ N; 21o23′ E; 1,340 m a.s.l.) in a mesotrophic grassland with a plant community that resembles the MG5 plant community *Cynosurus cristatus*-*Centaurea ni*gra grassland with a *Galium verum* sub-community [47]. Starting in spring 2004, two levels of N (0 and 15 g N m^−2^ year^−1^ applied as NH_4_NO_3_) and two levels of P (0 and 10 g m^−2^ year^−1^ applied as superphosphate) were applied annually on experimental plots (1.5×1.5 m) resulting in the formation of a factorial N and P fertilization experiment with eight blocks. Some details of the experimental site and analytical procedures have been described in Veresoglou et al. [44]; this previous manuscript described the microbial community shifts that resulted from fertilization practices. No specific permits were required for the described field study. The location was not privately-owned and was not designated as a protected area. Under Greek law no further permission was required to sample the area for scientific purposes. No endangered or protected species were involved in the field sampling.

### Soil Sampling and Analysis

On the 10th of July 2008 a harvest was carried out on the abovementioned site. Five blocks were randomly selected and in each of the twenty plots (5 blocks × 4 treatments) two soil cores (2 cm internal diameter, 8 cm depth) were retrieved, one centered on an *Agrostis capillaris* stand and one around a *Prunella vulgaris* individual. The cores were immediately frozen in liquid N_2_ and were subsequently freeze-dried, then following removal of visible plant material were ball milled for PLFA extraction. PLFA extraction was implemented according to White et al., [48] and analysis followed in a compound specific gas chromatograph-isotope ratio mass spectrometer GC-C-IRMS (GC Trace Ultra with combustion column attached via a GC Combustion III to a Delta V Advantage isotope ratio mass spectrometer Thermo Finnigan, Bremen, Germany). Samples (2 *µ*l) were injected in splitless mode onto a J&W Scientific HP-5 column, 50 m length, id 0.2 mm with a film thickness of 0.33 *µ*m (Agilent Technologies Inc, Santa Clara, USA); otherwise running conditions were as described by Paterson et al. [9]. The C isotope ratios were calculated with respect to a CO_2_ reference gas injected with every sample and traceable to International Atomic Energy Agency reference material NBS 19 TS-Limestone. Repeated analysis, over a two month period, of the δ^13^C value of a 19:0 FAME internal standard gave a standard deviation of 1.11 ‰ (*n* = 18).

### Data Analysis

Standard nomenclature was used for PLFAs [49] as follows: the number before the colon indicates the number of carbon atoms in the phospholipids and the number after the colon gives the number of double bonds and their location (ω). Prefixes Me, cy, i and a indicate presence of methyl- and cyclopropyl- groups and iso- or antesio- branching, respectively. The PLFA signatures 16:1ω7, 17:0cy, 19:0cy, 19:1w6, 15:0i, 15:0a, 16:0i, 17:0i, 17:0a 10-Me-17:0, 10-Me-18:0 were attributed to bacteria [23]. The fatty acid 18:2ω6,9 was used as a marker for fungi other than arbuscular mycorrhizal (AM) fungi [50] and the signatures 10-Me-17:0 and 10-Me-18:0 as markers for actinomycetes [51]. Signature 16:1ω5c was used as a biomarker of AM fungi [52].

Data were subjected to Kolmogorov-Smirnov tests for normality prior to analyses. To compute mean δ^13^C of microbial groups that included more than one PLFA signature, a weighted mean was obtained with individual PLFA signature PLFA concentrations. Analyses of variance were conducted based on three-way ANOVA models with factors P fertilization, N fertilization and plant identity. Hypothesis testing significance level was corrected with a classical Bonferroni procedure. Principal component analysis (PCA) was conducted in the standardized matrices of PLFA δ^13^C signals. The analysis was implemented with the package “vegan” in R 2.12.0 [53]. The number of significant components was retrieved following the broken-stick model [54]. PCA scores of the significant components were subjected to a MANOVA analysis in SPSS v. 15.0 (SPSS, Inc. IL: Chicago) with factors P fertilization, N fertilization and plant identity to test significance of experimental treatments. Presence of differences in variance amongst the fertilization treatments were investigated through (i) Hartley’s *F*max tests separately for the scores in each principal component [55], (ii) calculation of the size of the convex hulls of combinations of the significant components as performed in earlier studies [56], [57] and (iii) Mahalanobis distances [58] separately calculated for each sample that were analysed based on a Kruskal-Wallis test. Convex hull sizes were calculated with the function convexhull.xy in the package “spatstat” in R.

Finally, we tested significance of the correlation between the change in abundance of PLFAs and the change in their isotopic signal. We used as controls the PLFA δ^13^C data from the non-fertilized plots. For the three fertilization treatments, calculated separately for the two plants, the weighted difference in concentration with the respective non-fertilized control was calculated and the difference in signal, Δ^13^C approximated as Δ^13^C ≈ δ^13^C fertilized - δ^13^C control (21 signatures × 2 plants × 3 fertilization treatments = 126 points). Due to deviation from normality of both metrics Spearman’s rank test (Spearman’s rho or *ρ* test) was used.

## Supporting Information

Figure S1 **Heterogeneity in Moisture** **Content.** Means (±S.E) of moisture content of the samples obtained in the harvest of the 10^th^ of July 2008 grouped according to the blocking factor.
(DOC)

Table S1 **Mahalanobis-distances-based matrix.** Mahalanobis distances for the centroids of the samples following a principal component analysis. Analysis of the effect of the four fertilization treatments on the ranking of the Mahalanobis distances based on a Kruskal-Wallis test revealed significance of the effect of treatment.
(DOC)
