# Supplementary material for: Soil Fertilization Leads to a Decline in Between-Samples Variability of Microbial Community δ13C Profiles in a Grassland Fertilization Experiment
Source: PLoS One. 2012 Sep 4;7(9):e44203. doi: 10.1371/journal.pone.0044203 (PMC3433468; doi:10.1371/journal.pone.0044203)
Supplement: Figure S1 — Heterogeneity in Moisture Content. Means (±S.E) of moisture content of the samples obtained in the harvest of the 10th of July 2008 grouped according to the blocking factor. (DOC) [file pone.0044203.s001.doc]

Fig S1.
