# Supplementary material for: Soil Fertilization Leads to a Decline in Between-Samples Variability of Microbial Community δ13C Profiles in a Grassland Fertilization Experiment
Source: PLoS One. 2012 Sep 4;7(9):e44203. doi: 10.1371/journal.pone.0044203 (PMC3433468; doi:10.1371/journal.pone.0044203)
Supplement: Table S1 — Mahalanobis-distances-based matrix. Mahalanobis distances for the centroids of the samples following a principal component analysis. Analysis of the effect of the four fertilization treatments on the ranking of the Mahalanobis distances based on a Kruskal-Wallis test revealed significance of the effect of treatment. (DOC) [file pone.0044203.s002.doc]

| Table S1. Mahalanobis distances for the centroids of the samples following a principal component analysis. Analysis of the effect of the four fertilization treatments on the ranking of the Mahalanobis distances based on a Kruskal-Wallis test revealed significance of the effect of treatment. | | | | | | | | | | |
| --- | --- | --- | --- | --- | --- | --- | --- | --- | --- | --- |
| # | Block | Plant | N | P | PC1 | PC2 | PC3 | PC4 | PC5 | Mahalanobis |
| 1 | 1 | 0 | 0 | 0 | 0.63 | -0.36 | -0.26 | 0.04 | -0.66 | 12.03 |
| 2 | 2 | 0 | 0 | 0 | 0.14 | -0.22 | -0.12 | 0.07 | -0.36 | 3.22 |
| 3 | 3 | 0 | 0 | 0 | -0.11 | -0.53 | -0.07 | -0.11 | -0.06 | 3.20 |
| 4 | 4 | 0 | 0 | 0 | -0.42 | -0.09 | 0.49 | -0.68 | -0.23 | 12.05 |
| 5 | 5 | 0 | 0 | 0 | -0.57 | -0.03 | -0.06 | -0.24 | -0.30 | 3.99 |
| 6 | 1 | 0 | 1 | 0 | -0.60 | 0.51 | 0.23 | 0.17 | -0.32 | 7.07 |
| 7 | 2 | 0 | 1 | 0 | -0.26 | 0.20 | -0.11 | -0.15 | -0.18 | 1.80 |
| 8 | 3 | 0 | 1 | 0 | 0.66 | 0.24 | 0.50 | 0.56 | -0.18 | 10.95 |
| 9 | 4 | 0 | 1 | 0 | 0.55 | -0.18 | -0.08 | 0.39 | 0.10 | 4.27 |
| 10 | 5 | 0 | 1 | 0 | 0.39 | -0.02 | 0.17 | 0.05 | -0.16 | 1.48 |
| 11 | 1 | 0 | 0 | 1 | 0.24 | -0.25 | 0.06 | -0.32 | 0.02 | 2.58 |
| 12 | 2 | 0 | 0 | 1 | 0.04 | -0.24 | -0.29 | -0.07 | -0.38 | 4.35 |
| 13 | 3 | 0 | 0 | 1 | 0.81 | 0.10 | 0.35 | 0.28 | 0.12 | 5.79 |
| 14 | 4 | 0 | 0 | 1 | 0.22 | -0.19 | -0.14 | 0.03 | 0.08 | 0.93 |
| 15 | 5 | 0 | 0 | 1 | 0.14 | 0.11 | 0.25 | -0.40 | 0.09 | 3.69 |
| 16 | 1 | 0 | 1 | 1 | 0.28 | -0.13 | -0.17 | 0.13 | -0.13 | 1.43 |
| 17 | 2 | 0 | 1 | 1 | 0.46 | 0.04 | 0.04 | -0.29 | 0.02 | 2.27 |
| 18 | 3 | 0 | 1 | 1 | -0.06 | 0.37 | -0.03 | -0.04 | -0.12 | 1.71 |
| 19 | 4 | 0 | 1 | 1 | -0.09 | 0.22 | -0.39 | 0.10 | 0.24 | 3.66 |
| 20 | 5 | 0 | 1 | 1 | -0.57 | 0.14 | -0.20 | -0.11 | -0.11 | 2.47 |
| 21 | 1 | 1 | 0 | 0 | -1.91 | -0.29 | -0.46 | 0.26 | 0.08 | 19.24 |
| 22 | 2 | 1 | 0 | 0 | -0.80 | -1.00 | 0.90 | 0.28 | 0.30 | **25.58*** |
| 23 | 3 | 1 | 0 | 0 | -0.36 | 0.39 | 0.05 | -0.08 | 0.27 | 3.58 |
| 24 | 4 | 1 | 0 | 0 | 0.06 | 0.40 | 0.18 | -0.29 | 0.23 | 4.40 |
| 25 | 5 | 1 | 0 | 0 | -0.10 | 0.08 | 0.07 | 0.22 | 0.05 | 0.96 |
| 26 | 1 | 1 | 1 | 0 | -0.10 | 0.33 | -0.19 | 0.11 | 0.12 | 2.10 |
| 27 | 2 | 1 | 1 | 0 | -0.37 | 0.44 | 0.16 | 0.08 | -0.45 | 6.79 |
| 28 | 3 | 1 | 1 | 0 | -0.41 | 0.52 | -0.05 | 0.32 | 0.14 | 5.44 |
| 29 | 4 | 1 | 1 | 0 | 0.18 | 0.39 | -0.09 | -0.04 | 0.13 | 2.13 |
| 30 | 5 | 1 | 1 | 0 | -0.32 | 0.10 | 0.61 | 0.28 | -0.02 | 6.34 |
| 31 | 1 | 1 | 0 | 1 | -0.03 | -0.38 | -0.57 | 0.26 | 0.15 | 6.88 |
| 32 | 2 | 1 | 0 | 1 | -0.12 | -0.19 | -0.18 | -0.21 | 0.26 | 2.76 |
| 33 | 3 | 1 | 0 | 1 | 0.13 | -0.25 | -0.16 | 0.03 | 0.02 | 1.04 |
| 34 | 4 | 1 | 0 | 1 | 0.45 | 0.10 | -0.15 | -0.13 | 0.36 | 3.85 |
| 35 | 5 | 1 | 0 | 1 | 0.52 | -0.13 | -0.16 | 0.31 | 0.02 | 3.13 |
| 36 | 1 | 1 | 1 | 1 | 0.36 | -0.01 | -0.06 | -0.20 | 0.03 | 1.24 |
| 37 | 2 | 1 | 1 | 1 | 0.26 | -0.29 | 0.12 | -0.36 | 0.15 | 3.82 |
| 38 | 3 | 1 | 1 | 1 | 0.52 | -0.11 | -0.02 | -0.11 | 0.11 | 1.59 |
| 39 | 4 | 1 | 1 | 1 | 0.35 | -0.09 | -0.20 | -0.01 | 0.28 | 2.58 |
| 40 | 5 | 1 | 1 | 1 | -0.20 | 0.29 | 0.04 | -0.09 | 0.28 | 2.60 |
| *Highlighted value may represent an outlier according to the commonly applied chi-square criterion (the threshold is χ^2^(0.001, 5)= 20.5). However even following exclusion of the specific sample, the mean Mahalanobis distance in the controls (6.96) is considerably higher than in the other treatments (N: 4.84; P: 3.5; NP: 2.34). | | | | | | | | | | |
